# Supplementary material for: Evidence-based beta blocker use associated with lower heart failure readmission and mortality, but not all-cause readmission, among Medicare beneficiaries hospitalized for heart failure with reduced ejection fraction
Source: PLoS One. 2020 Jul 9;15(7):e0233161. doi: 10.1371/journal.pone.0233161 (PMC7347167; doi:10.1371/journal.pone.0233161)
Supplement: S1 Method — (DOCX) [file pone.0233161.s001.docx]

**S1 Methods**

ICD-9 codes that were used to define HF readmission were:

402.01 Malignant hypertensive heart disease with congestive heart failure (CHF)

402.11 Benign hypertensive heart disease with CHF

402.91 Hypertensive heart disease with CHF

404.01 Malignant hypertensive heart and renal disease with CHF

404.11 Benign hypertensive heart and renal disease with CHF

404.91 Unspecified hypertensive heart and renal disease with CHF

428.0 Congestive heart failure, unspecified

428.1 Left heart failure

428.20 Systolic heart failure, unspecified

428.21 Systolic heart failure, acute

428.22 Systolic heart failure, chronic

428.23 Systolic heart failure, acute or chronic

428.30 Diastolic heart failure, unspecified

428.31 Diastolic heart failure, acute

428.32 Diastolic heart failure, chronic

428.33 Diastolic heart failure, acute or chronic

428.40 Combined systolic and diastolic heart failure, unspecified

428.41 Combined systolic and diastolic heart failure, acute

428.42 Combined systolic and diastolic heart failure, chronic

428.43 Combined systolic and diastolic heart failure, acute or chronic

428.9 Heart failure, unspecified

- ***Anemia*:**

Any one of following:

- ≥1 inpatient, skilled nursing facility, or home health agency claim with any of following ICD-9 diagnosis codes (any position): 280.0x, 280.1x, 280.8x, 280.9x, 281.0x, 281.1x, 281.2x, 281.3x, 281.4x, 281.8x, 281.9x, 282.0x, 282.1x, 282.2x, 282.3x, 282.40, 282.41, 282.42, 282.43, 282.44, 282.45, 282.46, 282.47, 282.49, 282.5x, 282.60, 282.61, 282.62, 282.63, 282.64, 282.68, 282.69, 282.7x, 282.8x, 282.9x, 283.0x, 283.10, 283.11, 283.19, 283.2x, 283.9x, 284.01, 284.09, 284.11, 284.12, 284.19, 284.2x, 284.81, 284.89, 284.9x, 285.0x, 285.1x, 285.21, 285.22, 285.29, 285.3x, 285.8x, 285.9x.
- ≥1 outpatient or carrier file with any of following ICD-9 diagnosis codes (any position): 280.0x, 280.1x, 280.8x, 280.9x, 281.0x, 281.1x, 281.2x, 281.3x, 281.4x, 281.8x, 281.9x, 282.0x, 282.1x, 282.2x, 282.3x, 282.40, 282.41, 282.42, 282.43, 282.44, 282.45, 282.46, 282.47, 282.49, 282.5x, 282.60, 282.61, 282.62, 282.63, 282.64, 282.68, 282.69, 282.7x, 282.8x, 282.9x, 283.0x, 283.10, 283.11, 283.19, 283.2x, 283.9x, 284.01, 284.09, 284.11, 284.12, 284.19, 284.2x, 284.81, 284.89, 284.9x, 285.0x, 285.1x, 285.21, 285.22, 285.29, 285.3x, 285.8x, 285.9x linked to physician E&M code: hcpcs_cd in ('99024' '99058' '99429' '99499') or '99201'<=hcpcs_cd<='99288' or '99291'<=hcpcs_cd<='99292' or '99301'<=hcpcs_cd<='99337' or '99341'<=hcpcs_cd<='99357' or '99385'<=hcpcs_cd<='99387' or '99395'<=hcpcs_cd<='99404').
- ***Atrial fibrillation*:**

Any one of the following:

- ≥1 Inpatient claim with ICD-9 diagnosis code (any position) of 427.31.
- ≥2 claim from outpatient or carrier file on separate calendar days with ICD-9 diagnosis code (any position) of 427.31 linked to a physician E&M code.
- ***Chronic obstructive pulmonary disease (COPD):***

Any of the following:

- ≥1 inpatient, skilled nursing facility, or home health agency claim with any of following ICD-9 diagnosis codes (any position): 490, 491.0, 491.1, 491.8, 491.9, 492.0, 492.8, 491.20, 491.21, 491.22, 494.0, 494.1, or 496.
- ≥2 outpatient or carrier claims on separate calendar days with any of following ICD-9 diagnosis codes (any position): 490, 491.0, 491.1, 491.8, 491.9, 492.0, 492.8, 491.20, 491.21, 491.22, 494.0, 494.1, or 496 linked by physician E&M code.
- ***Depression*:**

Any one of the following:

- ≥1 inpatient, skilled nursing facility, home health agency claim with any of following ICD-9 diagnosis codes (any position): 296.20, 296.21, 296.22, 296.23, 296.24, 296.25, 296.26, 296.30, 296.31, 296.32, 296.33, 296.34, 296.35, 296.36, 296.51, 296.52, 296.53, 296.54, 296.55, 296.56, 296.60, 296.61, 296.62, 296.63, 296.64, 296.65, 296.66, 296.89, 298.0, 300.4, 309.1, or 311.
- ≥1 outpatient, or carrier claim with any of following ICD-9 diagnosis codes (any position): 296.20, 296.21, 296.22, 296.23, 296.24, 296.25, 296.26, 296.30, 296.31, 296.32, 296.33, 296.34, 296.35, 296.36, 296.51, 296.52, 296.53, 296.54, 296.55, 296.56, 296.60, 296.61, 296.62, 296.63, 296.64, 296.65, 296.66, 296.89, 298.0, 300.4, 309.1, or 311 linked to physician E&M code.
- ***Hypotension***

Any one of the following:

- ≥1 inpatient, skilled nursing facility, home health agency claim with ICD-9 diagnosis code (any position) of 458.xx.
- ≥1 outpatient, or carrier claim with ICD-9 diagnosis code (any position) of 458.xx linked to physician E&M code.
- ***Liver disease*:**

Any one of the following:

- ≥1 Inpatient claim with any of following ICD-9 diagnosis codes (any position): 070.0x, 070.1x, 070.20–070.23, 070.30–070.33, 070.41–070.44, 070.49, 070.51–070.54, 070.59, 070.6, 070.70, 070.71, 070.9x, 275.0x, 275.01–275.03, 275.09, 275.1x, 456.0x, 456.1x, 456.20, 456.21, 571.0x–571.3x, 571.40–571.42, 571.49, 571.5x, 571.6x, 571.8x, 571.9x, 572.2x–572.4x, 572.8x, 573.3x, 573.5x, 573.8x, 576.1x, 576.8x, 782.4x, E947.9, V02.60, V02.61, V02.62, V02.69, or V42.7x.
- ≥2 claim from carrier file or outpatient claims on separate calendar days with any of following ICD-9 diagnosis codes (any position): 070.0x, 070.1x, 070.20–070.23, 070.30–070.33, 070.41–070.44, 070.49, 070.51–070.54, 070.59, 070.6, 070.70, 070.71, 070.9x, 275.0x, 275.01–275.03, 275.09, 275.1x, 456.0x, 456.1x, 456.20, 456.21, 571.0x–571.3x, 571.40–571.42, 571.49, 571.5x, 571.6x, 571.8x, 571.9x, 572.2x–572.4x, 572.8x, 573.3x, 573.5x, 573.8x, 576.1x, 576.8x, 782.4x, E947.9, V02.60, V02.61, V02.62, V02.69, or V42.7x linked to a physician E&M code.
- ***Malnutrition:***

Any of the following:

- ≥1 inpatient claim with any of following ICD-9 diagnosis codes (any position): 262.xx, 263.8x, 263.9x, 799.4x, 263.0x-263.2x.
- ≥2 outpatient or carrier claims on separate calendar days with any of following ICD-9 diagnosis codes (any position): 262.xx, 263.8x, 263.9x, 799.4x, 263.0x-263.2x linked by physician E&M code.

**Implanted cardiac devices**

These were identified by one of the following

- any procedure code 0051, 0054, 3794, 3795, 3796, 3797, or 3798; or
- any cpt code 0319T, 0326T, 33230, 33231, 33240, 33249, 93282, 93283, 93284, 93287, 93289, 93295, 93640, 93641, 93642, G0291, or G0298
