# Supplementary material for: Evidence-based beta blocker use associated with lower heart failure readmission and mortality, but not all-cause readmission, among Medicare beneficiaries hospitalized for heart failure with reduced ejection fraction
Source: PLoS One. 2020 Jul 9;15(7):e0233161. doi: 10.1371/journal.pone.0233161 (PMC7347167; doi:10.1371/journal.pone.0233161)
Supplement: S1 Fig — (DOCX) [file pone.0233161.s002.docx]

**
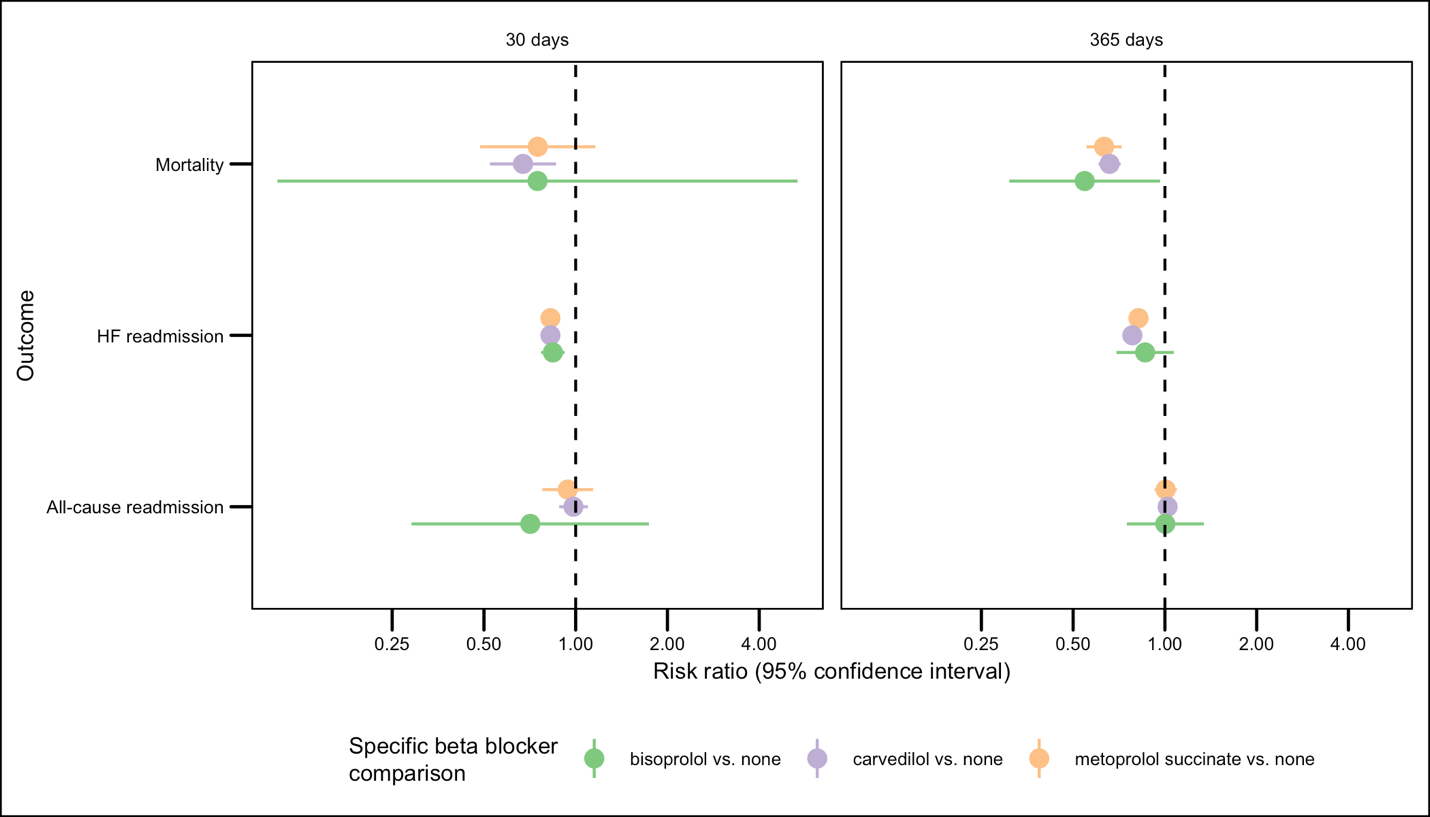
**

**Supplemental Figure 1. Risk ratios (RRs) and 95% confidence intervals for filling a prescription for a specific beta blocker (carvedilol, bisoprolol, or sustained-release metoprolol succinate) vs. no fill after discharge from a hospitalization for heart failure with reduced ejection fraction (HFrEF).** Models were adjusted for age at admission, sex, race, US census region, year of HFrEF hospitalization, as well as several variables assessed during the year prior to hospitalization: type of beta blocker use (evidence-based beta blocker for HFrEF, any other beta blocker, or none), ACEI/ARB use, diuretic use, dual-eligibility, Medicare Part D subsidy, nursing home residence, atrial fibrillation, malnutrition, liver disease, anemia, depression, COPD, Charlson comorbidity index, hospitalization, and a skilled nursing facility (SNF) stay. An RR of 1 indicated no significant association. Although confidence intervals are plotted, the intervals are so narrow that some are hard to see.
