# Supplementary material for: Evidence-based beta blocker use associated with lower heart failure readmission and mortality, but not all-cause readmission, among Medicare beneficiaries hospitalized for heart failure with reduced ejection fraction
Source: PLoS One. 2020 Jul 9;15(7):e0233161. doi: 10.1371/journal.pone.0233161 (PMC7347167; doi:10.1371/journal.pone.0233161)
Supplement: S1 Table — (DOCX) [file pone.0233161.s003.docx]

### **S1 Table. Summary of beneficiaries with a hospitalization for HFrEF between 2007 and 2013 who were and were not included in the current analysis.** Values are n(%) unless otherwise noted.

| **Variable** | **Level** | **Excluded from analysis sample^b^ (N=48,513)** | **Included in analysis sample (N=12,127)** |
| --- | --- | --- | --- |
| Age at admission (years)^a^ |  | 77.7 (12.1) | 75.1 (12.3) |
| Race | Black | 7,380 (15.2) | 1,891 (15.6) |
|  | Other | 3,083 (6.4) | 719 (5.9) |
|  | White | 38,050 (78.4) | 9,517 (78.5) |
| Women |  | 31,380 (64.7) | 5,970 (49.2) |
| Dual-eligible for Medicare and Medicaid |  | 22,515 (46.4) | 4,659 (38.4) |
| Medicare Part D subsidy |  | 25,802 (53.2) | 5,672 (46.8) |
| US Census region | East North Central | 8,284 (17.1) | 2,095 (17.3) |
|  | East South Central | 4,305 (8.9) | 1,265 (10.4) |
|  | Middle Atlantic | 6,830 (14.1) | 1,698 (14.0) |
|  | Mountain | 1,645 (3.4) | 476 (3.9) |
|  | New England | 2,705 (5.6) | 567 (4.7) |
|  | Pacific | 4,545 (9.4) | 1,122 (9.3) |
|  | South Atlantic | 10,254 (21.1) | 2,346 (19.3) |
|  | West North Central | 3,847 (7.9) | 941 (7.8) |
|  | West South Central | 6,098 (12.6) | 1,617 (13.3) |
| Anemia |  | 29,374 (60.5) | 6,527 (53.8) |
| Asthma |  | 8,542 (17.6) | 1,904 (15.7) |
| Atrial fibrillation |  | 22,138 (45.6) | 5,547 (45.7) |
| Atrioventricular block (2nd or 3rd degree) |  | 300 (0.6) | 77 (0.6) |
| Beta blocker use at baseline | Evidence-based | 18,910 (39.0) | 6,212 (51.2) |
|  | None | 14,202 (29.3) | 3,016 (24.9) |
|  | Other beta blocker | 15,401 (31.7) | 2,899 (23.9) |
| ACEI/ARB use |  | 31,229 (64.4) | 8,356 (68.9) |
| Diuretic use |  | 36,676 (75.6) | 9,143 (75.4) |
| Bradycardia |  | 940 (1.9) | 182 (1.5) |
| COPD |  | 24,862 (51.2) | 5,759 (47.5) |
| Cardiogenic shock |  | 127 (0.3) | 98 (0.8) |
| Charlson comorbidity index | 0 | 12,363 (25.5) | 4,618 (38.1) |
| Charlson comorbidity index | 1 - 3 | 6,042 (12.5) | 1,550 (12.8) |
| Charlson comorbidity index | >=4 | 30,108 (62.1) | 5,959 (49.1) |
| Depression |  | 12,405 (25.6) | 2,443 (20.1) |
| Discharged against medical advice |  | 269 (0.6) | 68 (0.6) |
| Discharged to hospice |  | 1,116 (2.3) | 499 (4.1) |
| Hospitalization during baseline |  | 13,112 (27.0) | 3,361 (27.7) |
| Hypotension |  | 8,418 (17.4) | 2,433 (20.1) |
| Liver disease |  | 2,379 (4.9) | 569 (4.7) |
| Malnutrition |  | 4,027 (8.3) | 822 (6.8) |
| Nursing home residence |  | 8,923 (18.4) | 871 (7.2) |
| Skilled nursing facility stay |  | 9,904 (20.4) | 1,432 (11.8) |
| Year of hospitalization | 2007 | 10,303 (21.2) | 615 (5.1) |
|  | 2008 | 8,027 (16.5) | 1,642 (13.5) |
|  | 2009 | 7,126 (14.7) | 1,823 (15.0) |
|  | 2010 | 6,374 (13.1) | 1,968 (16.2) |
|  | 2011 | 5,820 (12.0) | 1,988 (16.4) |
|  | 2012 | 5,414 (11.2) | 1,987 (16.4) |
|  | 2013 | 5,449 (11.2) | 2,104 (17.3) |

**^a^**mean(sd); ^b^Beneficiaries were excluded if they did not have a primary discharge diagnosis ICD-9 code of 428.2x or 428.4x, or if they were discharged to a skilled nursing facility.
