# Supplementary material for: Evidence-based beta blocker use associated with lower heart failure readmission and mortality, but not all-cause readmission, among Medicare beneficiaries hospitalized for heart failure with reduced ejection fraction
Source: PLoS One. 2020 Jul 9;15(7):e0233161. doi: 10.1371/journal.pone.0233161 (PMC7347167; doi:10.1371/journal.pone.0233161)
Supplement: S2 Table — (DOCX) [file pone.0233161.s004.docx]

**S2 Table. Summary of subsample similar to OPTIMIZE-HF by whether beneficiary filled a prescription for an evidence-based beta blocker within 30 days.** Values are n(%) unless otherwise specified. Some cell counts are suppressed with an “-“ if they were less than 11, due to privacy requirements from CMS.

| **Variable** | **Level** | **Filled prescription for beta blocker within 30 days (n = 668)** | **Did not fill prescription for beta blocker within 30 days (n = 945)** |
| --- | --- | --- | --- |
| Age at admission (years)^a^ |  | 78.9 (7.9) | 81.0 (8.4) |
| Race | Black | 85 (12.7) | 95 (10.1) |
|  | Other | 42 (6.3) | 49 (5.2) |
|  | White | 541 (81.0) | 801 (84.8) |
| Women |  | 362 (54.2) | 490 (51.9) |
| Dual-eligible for Medicare and Medicaid |  | 231 (34.6) | 291 (30.8) |
| Medicare Part D subsidy |  | 278 (41.6) | 366 (38.7) |
| US Census region | East North Central | 112 (16.8) | 144 (15.2) |
|  | East South Central | 64 (9.6) | 102 (10.8) |
|  | Middle Atlantic | 80 (12.0) | 129 (13.7) |
|  | Mountain | 38 (5.7) | 42 (4.4) |
|  | New England | 31 (4.6) | 43 (4.6) |
|  | Pacific | 73 (10.9) | 90 (9.5) |
|  | South Atlantic | 115 (17.2) | 176 (18.6) |
|  | West North Central | 64 (9.6) | 70 (7.4) |
|  | West South Central | 91 (13.6) | 149 (15.8) |
| Anemia |  | 270 (40.4) | 475 (50.3) |
| Atrial fibrillation |  | 220 (32.9) | 458 (48.5) |
| Atrioventricular block (2nd or 3rd degree) |  | - | - |
| ACEI/ARB use |  | 333 (49.9) | 439 (46.5) |
| Diuretic use |  | 361 (54.0) | 567 (60.0) |
| Bradycardia |  | - | 11 (1.2) |
| COPD |  | 237 (35.5) | 417 (44.1) |
| Charlson comorbidity index | 0 | 243 (36.4) | 331 (35.0) |
|  | 1 - 3 | 160 (24.0) | 167 (17.7) |
|  | >=4 | 265 (39.7) | 447 (47.3) |
| Depression |  | 105 (15.7) | 128 (13.5) |
| Hospitalization during baseline |  | 83 (12.4) | 177 (18.7) |
| Liver disease |  | 17 (2.5) | 33 (3.5) |
| Malnutrition |  | 29 (4.3) | 47 (5.0) |
| Nursing home residence |  | 31 (4.6) | 73 (7.7) |
| Skilled nursing facility stay |  | 36 (5.4) | 78 (8.3) |
| Year of hospitalization | 2007 | 28 (4.2) | 55 (5.8) |
|  | 2008 | 93 (13.9) | 146 (15.4) |
|  | 2009 | 110 (16.5) | 151 (16.0) |
|  | 2010 | 136 (20.4) | 173 (18.3) |
|  | 2011 | 101 (15.1) | 140 (14.8) |
|  | 2012 | 98 (14.7) | 154 (16.3) |
|  | 2013 | 102 (15.3) | 126 (13.3) |

^a^mean (standard deviation)
