# Supplementary material for: Evidence-based beta blocker use associated with lower heart failure readmission and mortality, but not all-cause readmission, among Medicare beneficiaries hospitalized for heart failure with reduced ejection fraction
Source: PLoS One. 2020 Jul 9;15(7):e0233161. doi: 10.1371/journal.pone.0233161 (PMC7347167; doi:10.1371/journal.pone.0233161)
Supplement: S3 Table — (DOCX) [file pone.0233161.s005.docx]

**S3 Table. Risk ratios (RRs) and 95% confidence intervals for filling a prescription for an evidence-based beta blocker (carvedilol, bisoprolol, or sustained-release metoprolol succinate) after discharge from a hospitalization for heart failure with reduced ejection fraction (HFrEF), among those in a subsample similar to the OPTIMIZE-HF cohort^a^.**

| **Outcome^b^** | **30 days follow up** | **365 days follow up** |
| --- | --- | --- |
| HF readmission | 0.95 (0.91 - 0.99) | 1.00 (0.90 - 1.10) |
| Readmission | 1.21 (0.92 - 1.60) | 1.02 (0.88 - 1.17) |
| Mortality | 0.70 (0.36 - 1.38) | 0.64 (0.52 - 0.79) |

^a^Excluded beneficiaries if: they had filled a prescription for any beta blocker in the previous year, were less than 65 years old, had bradycardia or atrioventricular block (2nd or 3rd degree) with no accompanying implanted cardiac device, had asthma, or had hypotension in the year prior to hospitalization, were missing discharge status, were discharged to hospice, were discharged against medical advice, were transferred to an acute care facility, or who had cardiogenic shock during hospitalization for HFrEF.

^b^Models were adjusted for age at admission, sex, race, US census region, year of HFrEF hospitalization, as well as several variables assessed during the year prior to hospitalization: type of beta blocker use (evidence-based beta blocker for HFrEF, any other beta blocker, or none), ACEI/ARB use, diuretic use, dual-eligibility, Medicare Part D subsidy, nursing home residence, atrial fibrillation, malnutrition, liver disease, anemia, depression, COPD, Charlson comorbidity index, hospitalization, and a skilled nursing facility (SNF) stay. An HR of 1 indicated no association.
