# Supplementary material for: Evidence-based beta blocker use associated with lower heart failure readmission and mortality, but not all-cause readmission, among Medicare beneficiaries hospitalized for heart failure with reduced ejection fraction
Source: PLoS One. 2020 Jul 9;15(7):e0233161. doi: 10.1371/journal.pone.0233161 (PMC7347167; doi:10.1371/journal.pone.0233161)
Supplement: S4 Table — (DOCX) [file pone.0233161.s006.docx]

### **S4 Table. Risk ratios (RRs) for readmission and all-cause mortality comparing those filling a prescription for an evidence-based beta blocker (carvedilol, bisoprolol, or sustained-release metoprolol succinate) after discharge from a hospitalization for heart failure with reduced ejection fraction (HFrEF), among those with no evidence-based beta blockers available upon hospital admission.**

| **Outcome^a^** | **30 days follow up** | **365 days follow up** |
| --- | --- | --- |
| HF readmission | 0.84 (0.83 - 0.86) | 0.82 (0.79 - 0.86) |
| Readmission | 0.99 (0.88 - 1.10) | 1.02 (0.96 - 1.07) |
| Mortality | 0.69 (0.54 - 0.88) | 0.68 (0.62 - 0.74) |

^a^Models were adjusted for age at admission, sex, race, US census region, year of HFrEF hospitalization, as well as several variables assessed during the year prior to hospitalization: type of beta blocker use (evidence-based beta blocker for HFrEF, any other beta blocker, or none), ACEI/ARB use, diuretic use, dual-eligibility, Medicare Part D subsidy, nursing home residence, atrial fibrillation, malnutrition, liver disease, anemia, depression, COPD, Charlson comorbidity index, hospitalization, and a skilled nursing facility (SNF) stay. An HR of 1 indicated no association.
