# Supplementary material for: Evidence-based beta blocker use associated with lower heart failure readmission and mortality, but not all-cause readmission, among Medicare beneficiaries hospitalized for heart failure with reduced ejection fraction
Source: PLoS One. 2020 Jul 9;15(7):e0233161. doi: 10.1371/journal.pone.0233161 (PMC7347167; doi:10.1371/journal.pone.0233161)
Supplement: Attachment — Submitted filename: Response to Reviewers.docx [file pone.0233161.s008.docx]

**Response to Reviewers**

Associate editor

***Important points to address are the consideration of adjusting the analysis for other drugs,***

We thank the AE for these overarching and general comments. Concerning adjustment of the models for other drugs, which was noted also by Reviewer #2, we have included use of ACEI , ARB, and diuretic use in all statistical models. Even though the results changed only the hundredths decimal place, we believe that now *knowing* that our inferences did not change with the addition of these drugs to the model is highly valuable to the reader. We regret not adjusting for these drugs from the beginning

***the discussion based on current treatment guidelines***

We thank the editor for noting our omission of the recent updated treatment guidelines (Yancy et al. 2016, Yancy et al. 2017), which specifically address ARNIs and ivabradine. We have now added that omission to our limitations, given the time period during which our retrospective cohort study was conducted.

Yancy CW, Jessup M, Bozkurt B, Butler J, Casey DE Jr, Colvin MM, et al. 2016 ACC/AHA/HFSA Focused Update on New Pharmacological Therapy for Heart Failure: An Update of the 2013 ACCF/AHA Guideline for the Management of Heart Failure: A Report of the American College of Cardiology/American Heart Association Task Force on Clinical Practice Guidelines and the Heart Failure Society of America. J Am Coll Cardiol. 2016;68:1476–88.

Yancy CW, Jessup M, Bozkurt B, Butler J, Casey DE, Colvin MM, et al. 2017 ACC/AHA/HFSA focused update of the 2013 ACCF/AHA guideline for the management of heart failure: a report of the American College of Cardiology/American Heart Association Task Force on Clinical Practice Guidelines and the Heart Failure Society of America. J Am Coll Cardiol. Journal of the American College of Cardiology; 2017;70:776–803.

We have added the following text to our “Limitations” paragraph:

*First, because of the time period of our study, we were not able to assess the impact of newly recommended angiotensin receptor-neprilysin inhibitors (ARNI) or sinoatrial node modulators (i.e., ivabradine) in Medicare beneficiaries. Additional research with sufficient follow up time of Medicare beneficiaries using these medications will be critical for understanding these medications’ population-based impact.*

***and the appropriate discussion of the limitations.***

We will note our limitations more clearly.

***In addition, I am wondering if the authors provide information as to whether there was a shift from less HF hospitalizations to an increase in non-HF hospitalizations and if yes, which causes of hospitalization increased,***

In our models, we did adjust for year of hospitalization, since we observed a large jump in the number of hospitalizations in the Medicare database from 2007 to 2008. This jump was not due to a change in the true incidence of HFrEF, but due to changes in CMS reimbursement policies allowing *type* of HF to be noted. Therefore, the estimated association between beta blocker use and outcomes in our manuscript applies to each of the years under observation (2007 – 2014).

***or if the lack of effect on all-cause hospitalization was simply because non-HF / non-cardiac hospitalizations were much more common that the effect on HF-hospitalization was diluted.***

We share this conclusion with the AE, and we thank the AE for this astute point. Previous work from our group has shown that hospitalizations for HF accounted for only 1/3 of all causes of 30-day readmissions among Medicare beneficiaries hospitalized with HFrEF (Goyal et al. 2018). We will provide greater emphasis and clarity on this conclusion.

Goyal P, Loop M, Chen L, Brown TM, Durant RW, Safford MM, et al. Causes and Temporal Patterns of 30‐Day Readmission Among Older Adults Hospitalized With Heart Failure With Preserved or Reduced Ejection Fraction. J Am Heart Assoc [Internet]. 2018 [cited 2018 Aug 27]; Available from: https://www.ahajournals.org/doi/full/10.1161/JAHA.117.007785

Reviewer #1

***General comment, summary
The manuscript entitled “Evidence-based beta blocker use associated with lower heart failure readmission and mortality, but not all-cause readmission, among Medicare beneficiaries hospitalized for heart failure with reduced ejection fraction” by Loop et al. reported from a
retrospective cohort study of beneficiaries in the Medicare 5% sample hospitalized for
HFrEF between 2007 and 2013 and were discharged alive. They compared in total 12,127 participants, the 30-day and 365-day heart failure (HF) readmission, all-cause readmission, and mortality rates between beneficiaries who filled a prescription for an evidence-based beta blocker (carvedilol, bisoprolol, and sustained-release metoprolol succinate) and those who did not after being hospitalized for HFrEF.
The main finding of the analyses (adjusted risk models) and conclusion accordingly, was that increasing the use of beta blockers following HFrEF hospitalization is unlikely to decrease all-cause readmissions among Medicare beneficiaries, but may reduce HF-specific readmissions and mortality.

In my perspective, this analysis from a large HFrEF cohort, though retrospective, is well-conducted and the results are clinically important and add to our understanding of relevant outcomes associated with evidence-based HF therapies in real-world practice, e.g. in US.
The authors also included two sensitivity analyses in their report.
The present manuscript is easy-to-follow, well written, both in wording and grammar.
The comprehensive tables and figure, respectively contribute to the clear presentation of the results and interpretation.***

We thank the reviewer for these kind words about our research, and we are glad the paper was easy to follow and understand.

***Major comments
1. This retrospective cohort study is observational with its inherent limitations, although is a sample of real-world HF care in the US. The cohort, however, is not that contemporary.***

The reviewer is correct that the cohort is not that contemporary, now that the last date of follow up was approximately 7 years ago. We will add this limitation to our Discussion section and acknowledge it appropriately. Given that there have been no new evidence-based beta blocker therapies for HFrEF introduced since the end of follow up, we also believe our data still represents some of the best evidence concerning the association between these therapies and important outcomes among Medicare beneficiaries.

However, we were indeed not able to assess the impact of ARNI or ivabradine. Therefore, we have added the following statement to our “Limitations” paragraph:

*First, because of the time period of our study, we were not able to assess the impact of newly recommended angiotensin receptor-neprilysin inhibitors (ARNI) or sinoatrial node modulators (i.e., ivabradine) in Medicare beneficiaries. Additional research with sufficient follow up time of Medicare beneficiaries using these medications will be critical for understanding these medications’ population-based impact.*

***2. Definition of HFrEF is not clear, uniform, or at discretion of the physician. Although, the sample is random and for that reason, close to generalizability.***

As noted by the reviewer, we are relying on billing codes for HFrEF that are based on clinical care provided in a wide variety of settings and doctors across the United States. The diagnosis of the syndrome HFrEF is difficult and non-standard. We concur that using the 5% random sample of Medicare beneficiaries would guarantee that are results are generalizable to US Medicare beneficiaries diagnosed with HFrEF.

***3. Information on optimal or maximal beta-blocker doses is not provided (or lacking, not collected?); suboptimal up-titrated medication may be associated with more severe HFrEF, comorbidity burden, high age, and many other factors; and has effects on important morbidity and mortality.***

The reviewer makes an excellent point that we did not account for the dose of beta blocker prescription filled or to what degree that prescribed dose differed from the optimal/maximum tolerable dose.

In a previous paper, our group found that: (1) almost no Medicare beneficiaries are ever uptitrated with 1 year of hospitalization for heart failure; and (2) diagnosis of relative contraindications have surprisingly small associations with the probability of being uptitrated (Loop et al. 2018). Therefore, there is not a large amount of variation among participants that would prevent them from being uptitrated. We also found that some variables were consistently associated with receiving a dose for any of the 3 evidence-based beta blockers, leading us to include those comorbidities in the models for our current analysis (atrial fibrillation, malnutrition, liver disease, and anemia).

Loop MS, van Dyke MK, Chen L, Safford MM, Kilgore ML, Brown TM, et al. Low Utilization of Beta-Blockers Among Medicare Beneficiaries Hospitalized for Heart Failure With Reduced Ejection Fraction. J Card Fail [Internet]. 2018; Available from: http://dx.doi.org/10.1016/j.cardfail.2018.10.005

***4. Confounding by indication could only be partially accounted for.***

The reviewer has noted the largest limitation of this study. Even with our extensive set of adjustment factors that were chosen based upon evidence from previous research in the same database, we clearly did not adjust for all factors leading to filling a prescription for an evidence-based beta blocker. Information on severity of HFrEF and comorbidities and patient function and preference are not captured in claims data. We have placed more emphasis on this limitation, and its impact on the interpretation, in our Discussion section, as section of which now reads as follows:

*Perhaps the most consequential limitation of our study was that we could not fully account for the propensity of the sickest patients with HFrEF to not receive treatment (i.e., “confounding by indication”)* [*[23]*](https://paperpile.com/c/jI6lJO/A94v)*, due to lack of access to vital signs, laboratory measures, cardiac function (e.g., ejection fraction), and patient preference in Medicare data to calculate and adjust for an appropriate risk score* [*[24]*](https://paperpile.com/c/jI6lJO/CdlL)*. This may lead to an overestimation of the apparent benefits of beta blockers.*

***5. Do the authors have insights or due thoughts on (substantially reported) non-presciption of beta-blockers?***

It is not entirely clear whether the low rates of fills is due to low prescription or low fill rates. Unfortunately, prescriptions by the physician are not included in Medicare data, so we could not address these two pieces of fill rates separately.

Based on a small qualitative study we performed of 19 primary care physicians, the most common perceived barrier to use of beta blockers were treatment side effects, in particular hypotension (Levitan et al. 2017). These results somewhat conflict with our analysis of the Medicare 5% random sample, which reported that having diagnosis claims for contraindications such as hypotension had only small associations with filling a prescription for an evidence-based beta blocker. However, the discussions with primary care physicians indicated that they were concerned not only with existing conditions, but also with the potential for inducing problems such as hypotension and syncope.

Levitan EB, Van Dyke MK, Loop MS, O’Beirne R, Safford MM. Barriers to Beta-Blocker Use and Up-Titration Among Patients with Heart Failure with Reduced Ejection Fraction. Cardiovasc Drugs Ther. 2017;31:559–64.

***6. Differential analyses of each beta-blocker may add to our knowledge, e.g. carvedilol as additional pharmocodynamic properties.***

We thank the reviewer for this suggestion, as it was not an analysis we had considered. After considering the suggestion, we agreed that this sensitivity analysis was important and would be helpful to the reader. For the reviewer’s convenience, we reproduce below sections of the Methods and Results that describe this sensitivity analysis.

*Methods: … Fourth, we modified the exposure from “filled or not filled” to “filled carvedilol, filled bisoprolol, filled sustained-release metoprolol succinate, or not filled.” The first fill for one of these beta blockers determined the exposure category in which the beneficiary remained, regardless of future beta blocker fills. This sensitivity analysis attempted to detect any variation in the associations between fills for specific beta blockers and the outcomes of interest.*

*Results: …* *The final sensitivity analysis, which specified which particular evidence-based beta blocker was filled (carvedilol, bisoprolol, metoprolol succinate, or none), showed nearly identical HRs for the associations of filling for each specific beta blocker vs. no fill (Supplemental Figure 1). The confidence intervals for the HR for bisoprolol fill vs. no fill were sometimes much larger than for the other beta blockers, indicating fewer beneficiaries filling prescriptions for bisoprolol compared to the carvedilol or sustained-release metoprolol succinate.*

***Supplemental Figure 1. Risk ratios (RRs) and 95% confidence intervals for filling a prescription for a specific beta blocker (carvedilol, bisoprolol, or sustained-release metoprolol succinate) vs. no fill after discharge from a hospitalization for heart failure with reduced ejection fraction (HFrEF).*** *Models were adjusted for age at admission, sex, race, US census region, year of HFrEF hospitalization, as well as several variables assessed during the year prior to hospitalization: type of beta blocker use (evidence-based beta blocker for HFrEF, any other beta blocker, or none), ACEI/ARB use, diuretic use, dual-eligibility, Medicare Part D subsidy, nursing home residence, atrial fibrillation, malnutrition, liver disease, anemia, depression, COPD, Charlson comorbidity index, hospitalization, and a skilled nursing facility (SNF) stay. An RR of 1 indicated no significant association. Although confidence intervals are plotted, the intervals are so narrow that some are hard to see.*

***7. Do the authors have data on repeat hospitalizations?***

We initially did include both incident and repeat readmissions in our analysis, with adjustment for the correlated failure times within the same beneficiary. Although not presented in our current version of the manuscript, the results were largely identical. We felt that presenting the results for only the first observed event was more straightforward to explain and understand.

***8. Do the authors have data on (CIED) device usage; which may have effects on clinical outcomes? This also goes for non-HF cardiovascular medication.***

We did have access to procedure code data, indicating whether a participant had a pacemaker and/or defibrillator. After considering the reviewer’s question, we decided to include an additional sensitivity analysis that excluded individuals with implanted cardiac devices. We have reproduced sections from the Methods and Results below for the reviewer’s convenience.

*Methods: …* *Third, we repeated the analysis in a subset of participants who did not have history of a procedure code for an implanted cardiac device (pacemaker and/or defibrillator). Potential contraindications or variation in effects of beta blockers between those with and without an implanted cardiac device motivated this analysis.*

*Results: …* *The sensitivity analysis that excluded participants with an implanted cardiac device produced estimates almost identical to the main analysis as well (Supplemental Table 5).*

### ***Supplemental Table 5. Hazard ratios (HRs) for readmission and all-cause mortality comparing those filling a prescription for an evidence-based beta blocker (carvedilol, bisoprolol, or sustained-release metoprolol succinate) after discharge from a hospitalization for heart failure with reduced ejection fraction (HFrEF), among those with no implanted cardiac device.***

| ***Outcome^a^*** | ***30 days follow up*** | ***365 days follow up*** |
| --- | --- | --- |
| *HF readmission* | *0.85 (0.83 - 0.86)* | *0.83 (0.80 - 0.87)* |
| *Readmission* | *1.00 (0.90 - 1.12)* | *1.02 (0.96 - 1.08)* |
| *Mortality* | *0.70 (0.55 - 0.89)* | *0.65 (0.60 - 0.71)* |

*^a^Models were adjusted for age at admission, sex, race, US census region, year of HFrEF hospitalization, as well as several variables assessed during the year prior to hospitalization: type of beta blocker use (evidence-based beta blocker for HFrEF, any other beta blocker, or none), ACEI/ARB use, diuretic use, dual-eligibility, Medicare Part D subsidy, nursing home residence, atrial fibrillation, malnutrition, liver disease, anemia, depression, COPD, Charlson comorbidity index, hospitalization, and a skilled nursing facility (SNF) stay. An HR of 1 indicated no association.*

***9. How do the authors explain their finding that all-cause hospitalization is not significantly associated with filled prescription of a disease-modifying beta-blocker? In contrast to OPTIMIZE-HF, although in which all beta-blockers were taken into analysis?***

In the final paragraph of the Discussion section of the original manuscript, we stated the following: “Medicare beneficiaries might be readmitted for non-HF related conditions so often that beta blockers do not have a significant benefit on readmissions, even though beta blockers improve heart function [[19]](https://paperpile.com/c/jI6lJO/Ep93).” In our revised version of the manuscript, we have expanded this section to include the following text:

*Medicare beneficiaries might be readmitted for non-HF related conditions so often that beta blockers do not have a significant benefit on readmissions, even though beta blockers improve heart function* [*[17]*](https://paperpile.com/c/jI6lJO/Ep93)*. In both OPTIMIZE-HF [18] and our study population [19], approximately half of readmissions before 30 days were cardiovascular-related. We found that 20% of readmissions were non-HF cardiovascular readmission. Although the three beta blockers we studied are preferred in HFrEF because of their HFrEF-specific benefits, the other beta blockers may have beneficial effects on other non-HF cardiovascular causes of hospitalization. This possibility could help to explain the differences in finding between our study and OPTIMIZE-HF.*

We hope that the new text puts appropriate emphasis on our conclusions concerning why we saw RRs near 1 for all-cause readmission.

***10. The latter finding is strikingly important, is even incorporated in the manuscript title, and needs more detailed clarification and discussion accordingly, and guidance for future studies.***

We hope the additional text we provided in response to #9 above will satisfy this comment.

***Minor comments
1. What definitions of anemia, atrial fibrillation and other comorbidities were used?***

We apologize for this oversight in not including the definitions of comorbidities. We have corrected this error and included the ICD-9 codes used to define each of these comorbidities in the Supplemental Methods.

***2. The authors used claim for a prescription fill as a proxy for beta-blocker use by HFrEF patients; has that been validated previously?***

We are not aware of any rigorous validation studies that have determined the properties of using a prescription fill as a proxy for beta blocker use (i.e., swallowing the amount of medication at the time prescribed on the label). Such a validation study might be difficult, given that attempting to watch whether someone actually takes the pills might create some “observer bias” and cause people to take the medication more often than they normally would. Although we do not have a quantitative understanding of the validity of this proxy, we are relying on the qualitative improvement of our variable over presence of prescription for a beta blocker in the medical record. We believe it is reasonable to assume that claims for filling a prescription for a beta blocker, which incorporates some patient behavior above and beyond the provider behavior of noting a prescription for a beta blocker in the medical record, have less exposure misclassification error than using the presence of a prescription for a beta blocker in the medical record, such as in the discharge summary.

***3. There is no certainty at all for continued use of beta-blocker, discontinuation, or intolerance, and also, no reported reasons for modified medication regimens***

The reviewer is absolutely correct in pointing out this limitation. Although we had records of all beta blocker fills for each participant, we considered someone a beta blocker “user” after their first fill, thus aggregating together patients who continued to use them, patients who stopped filling prescriptions because of side effects, and patients who stopped filling prescriptions because their physician changed the prescription. Given the complexity of the time-varying exposure and time-varying hazard ratio models, we have chosen to make this simplifying assumption.

We have clarified in our Methods and Limitations sections that the two groups we are comparing are “those who will end up filling a prescription at least once within a year” to those who “do not fill a prescription within one year,” and that we have grouped several types of patients into one of the exposure groups. We thank the reviewer for emphasizing how large this assumption is, and we hope our next text has clarified that for the reader. The new text is reproduced below for the reviewer’s convenience.

*Methods: …* *Therefore, we used a claim for filling a prescription for an evidence-based beta blocker for HFrEF as a proxy for taking an evidence-based beta blocker at least once within a year following hospitalization.*

*Discussion: We assumed that filling a prescription for a beta blocker was a proxy for continued use throughout the period of follow up, but some participants will have stopped taking the medication during follow up in response to potential side effects or new contraindications. Therefore, out estimates of benefit may be exaggerated or underestimated compared to the true benefit.*

***4. Relatively more patients from lower socio-economic status were excluded, which may influence outcomes and also elaborated risk stratification and generalizability.***

We thank the reviewer for this comment, and we absolutely agree with it. In our manuscript, we note this limitation in the Discussion:

*Finally, even though our study targeted a more general patient population than previous RCTs of beta blockers or registry studies, we were still somewhat limited in our ability to generalize our results to all Medicare beneficiaries. Participants who were excluded from the study sample were more likely to be female, live in a nursing home, and have had a SNF stay in the year prior to hospitalization and less likely to be taking an evidence-based beta blocker, which were all expected differences given our exclusion criteria (see Supplemental Table 1). However, beneficiaries excluded from our analysis were also more likely to be dual-eligible for Medicare and Medicaid, have a Medicare Part D subsidy, and have a higher comorbidity burden. We therefore implicitly excluded sicker beneficiaries of lower socioeconomic status, and it is possible that these beneficiaries would have been less likely to fill a prescription for a beta blocker and have higher risk of the outcomes of interest. It is unclear whether our estimates generalize to these beneficiaries.*

***5. The authors may describe strengths and limitations in a more separate paragraph or section at the end of Discussion, just prior to Conclusions.***

We thank the reviewer for this suggestion. We have separately labeled our strengths and limitations into a labeled subsection, just before the Conclusion section. We think these points are now easier for the reader to identify and digest.

***6. In Figure 1, first panel, CIs are not depicted.***

We thank the reviewer for giving our paper a very careful look. There actually are confidence intervals depicted in the left-most panel of Figure 1(i.e., the “HF readmission” panel), but the uncertainty is so small that they do not appear beyond the width of the dots. We have updated the caption to this figure to include a statement about this issue.

Reviewer #2

***Summary:
This retrospective study investigated the effect of beta-blocker treatment in HFrEF beneficiaries in the Medicare 5% sample. For study inclusion, HFrEF patients had to have HFrEF related HF hospitalized between 2007 and 2013 and had to be alive at discharge.
Beta-blocker treatment after hospital discharge was assumed on the basis that study participants (overall 35%) filled prescription of an evidenced-based beta blocker (metoprolol, bisoprolol, carvedilol). These study participants were compared to study participants who did not fill a prescription for beta blocker treatment after hospital discharge. Outcome measure was 30 day and 365 day heart failure or all cause readmission rate, or all cause mortality. The first occurring event was always entered into the analysis. Out of 12127 beneficiaries with index hospitalization for HFrEF 30% were readmitted for HF hospitalization, 62% for any cause; 27% died within 365 days.
Filling a prescription for a beta blocker was associated with a lower HF readmission rate and a lower mortality rate but did not change the all-cause readmission rate in competing risk models.

Critique:
This retrospective analysis of data derived from U.S. medicare beneficiaries shows beneficial effects of beta-blocker treatment on heart failure readmission and mortality when study participants filled the beta blocker prescription within 0-3 day, 4-7 days, or > 7 days. This beneficial effect was already present within the first 30 days and persisted thereafter. This observation is surprising since Kaplan-Meier curves presenting survival or the combined endpoint of survival and heart-failure related hospitalization separated only after 3 months beta blocker treatment in the MERIT-HF or the COPERNICUS trial. Likewise, separation of survival curves in the BIOSTAT-CHF study occurred only after 100 days in patients on guidelines-based medical therapy. I wonder whether this important early effect is really associated with beta blocker treatment alone and not related to overall good compliance to drug treatment in the 35% of study participants filling the prescription?***

The reviewer has raised an excellent point. Indeed, our results show what we believe to be an unrealistically large benefit of beta blockers early on in follow up, which levels off after 8 days. We believe that early benefit was due to at least 2 factors: (1) anti-arrhythmic effects of beta blockers; and (2) confounding by indication (i.e., the sickest patients may not receive or fill prescriptions for beta blockers).

The reviewer’s comment was mostly focused on the effect seen within 30 days, in contrast to effects of similar magnitude seen at 90 days in randomized controlled trials and other observational studies. The reviewer suggested one difference between patients who filled their prescription and patients who did not was overall better compliance to drug treatment in general. We agree with the reviewer that this possibility is entirely reasonable, and we should have thought of it ourselves. We have added it to the Discussion section and reproduced the text below for the reviewer’s convenience.

*Another source of confounding could have been that patients who are more likely to be compliant to any drug regimen for HFrEF and thus have better outcomes may be more likely to fill a prescription for a beta blocker.*

***The authors should discuss why the results of this retrospective analysis are still valid in 2019. HFrEF treatment changed because of results of EMPHASIS-HF published in 2011 and the PARADIGM-HF published in 2015. Why did the authors choose to analyze the effect of beta blocker treatment on 365 days all-cause mortality in real-world patients 2007-2013?***

Analysis of data several years old should always be justified, and we regret not including a better justification in the original manuscript. We have added text to the Introduction to justify why our analysis has clinical and health outcomes implications in 2020.

We appreciate that the last several years have produced important breakthroughs in the treatment of HFrEF. In addition to the landmark EMPHASIS-HF (eplerenone) and PARADIGM-HF (sacubitril/valsartan), ivabradine was approved for sale in the United States in 2015. However, uptake of these agents has been very slow in this population. For example, in one US study (Sangaralingham et al. 2018), <3% of individuals with HFrEF used sacubitril/valsartan. Despite the introduction of new agents for HFrEF, the treatment landscape has not changed dramatically. Therefore, we think the results in this manuscript remain relevant.

Sangaralingham LR, Sangaralingham SJ, Shah ND, Yao X, Dunlay SM. Adoption of Sacubitril/Valsartan for the Management of Patients With Heart Failure. Circ Heart Fail. 2018;11:e004302.

We are placing the new text in the Introduction below for the Reviewer’s convenience.

*Additional therapies such as eplerenone [8] and sacubitril/valsartan [9] have demonstrated effective reduction in mortality and readmissions, but sacubitril/valsartan had yet to experience meaningful uptake in its first 18 months of approval through the end of 2016 [10]. Beta blocker therapy, in addition to other therapies such as ACE-inhibitors, remain common pharmaceutical therapies for HFrEF in the general patient population.*

***Why did the authors decide to measure the first hospitalization independent whether it was a hospitalization for heart failure or other reason? Reason for this question is the fact that it is clear that beta-blocker treatment should reduce heart failure related-hospitalization but not hospitalization for other comorbidity.***

We agree that beta blockers are most likely to reduce risk of hospitalization for HF and perhaps other cardiovascular disease. In the US Medicare system, all-cause readmission has been a major target of intervention and policy. Additionally, from a patient perspective, being sick enough to be hospitalized is a bad outcome. A medication that reduces one type of hospitalization without reducing overall hospitalization may not be valued sufficiently to cope with the costs and side effects.

***How can You exclude that the effect of beta-blocker treatment on heart failure related hospitalization was underestimated?***

Although we cannot exclude the possibility that the effect of beta blocker treatment was underestimated, we believe overestimation of the effect was a more likely scenario. Prior research has shown that healthier cardiovascular disease patients receive more intensive therapy. This preferential treatment can lead to overestimation of the apparent effects of these therapies. As noted in the manuscript, we have used a competing risk analysis to avoid overestimation of the association.

***Why did the authors did not adjust for other heart failure drug treatment?***

We thank the author for this question. After reconsidering our approach, we decided to adjust all models for ACEI/ARB use and diuretic use in the year prior to the hospitalization. We think that these medications are certainly a predictor of our outcomes of interest, as well as possible confounders of the association between beta blocker use and the outcomes, since beneficiaries filling a prescription for another heart failure medication may be more likely to fill prescriptions for beta blockers. These adjustments to all analyses have strengthened the manuscript, and we appreciate the Reviewer’s suggestion.
