# Supplementary material for: Evidence-based beta blocker use associated with lower heart failure readmission and mortality, but not all-cause readmission, among Medicare beneficiaries hospitalized for heart failure with reduced ejection fraction
Source: PLoS One. 2020 Jul 9;15(7):e0233161. doi: 10.1371/journal.pone.0233161 (PMC7347167; doi:10.1371/journal.pone.0233161)
Supplement: Attachment — Submitted filename: Response to Reviewers.docx [file pone.0233161.s009.docx]

April 23, 2020

Hans-Peter Brunner-La Rocca, M.D.

Academic Editor

PLOS ONE

Dr. Brunner-La Rocca,

Thank you very much for the opportunity to revise and resubmit our manuscript. Upon further considering of the comments from one reviewer and yourself, we whole-heartedly agree that our inability to separate the benefits of beta blockers from general adherence, especially to other therapies, is a limitation. Therefore, we have added a statement regarding this limitation in the Discussion, as well as in our Conclusions. We have reproduced both sets of text below for your convenience.

***Discussion***

*…*

*Another source of confounding could have been that patients who are more likely to be adherent to any drug regimen for HFrEF and thus have better outcomes may be more likely to fill a prescription for a beta blocker. Therefore, this analysis may overestimate the apparent benefits of beta blocker use. In particular, filling a prescription for a beta blocker shortly after discharge may be a marker of adherence to medical therapy in general, which may help explain the large apparent benefit on mortality immediately following hospitalization.*

***Conclusions***

*However, in this retrospective observational study, we were unable to disentangle the effects of beta blocker use from other characteristics and behaviors such as adherence to other therapies. This adherence to other therapies may have led to overestimation of the protective associations of beta blocker use, particularly on mortality.*

We very much hope these changes are satisfactory, as we would be proud to have this manuscript published in PLOS ONE.

Sincerely,

Matthew Shane Loop, PhD FAHA
